# Dicaesium tetra­magnesium penta­kis­(carbonate) deca­hydrate, Cs_2_Mg_4_(CO_3_)_5_·10H_2_O

**DOI:** 10.1107/S2414314620001650

**Published:** 2020-02-11

**Authors:** Christine Rincke, Horst Schmidt, Wolfgang Voigt

**Affiliations:** a Institute of Inorganic Chemistry, TU Bergakademie Freiberg, Leipziger Str. 29, D-09599 Freiberg, Germany; Vienna University of Technology, Austria

**Keywords:** carbonate, hydrate, magnesium compound, caesium compound, twinning, crystal structure

## Abstract

The title carbonate hydrate, Cs_2_Mg_4_(CO_3_)_5_·10H_2_O, was crystallized at room temperature out of aqueous solutions containing caesium bicarbonate and magnesium nitrate. Its monoclinic crystal structure (*P*2_1_/*n*) consists of double chains of composition ^1^_∞_[Mg(H_2_O)_2/1_(CO_3_)_3/3_], isolated [Mg(H_2_O)(CO_3_)_2_]^2–^ units, two crystallographically distinct Cs^+^ ions and a free water mol­ecule. The crystal under investigation was twinned by reticular pseudomerohedry.

## Abstract 2

The title compound, which formed twinned crystals, represents a highly hydrated carbonate with an unprecedented composition. Its unique crystal structure consists of a complex arrangement of anionic building units centred by octa­hedrally coordinated magnesium ions, caesium cations and free water mol­ecules.

## Graphical abstract

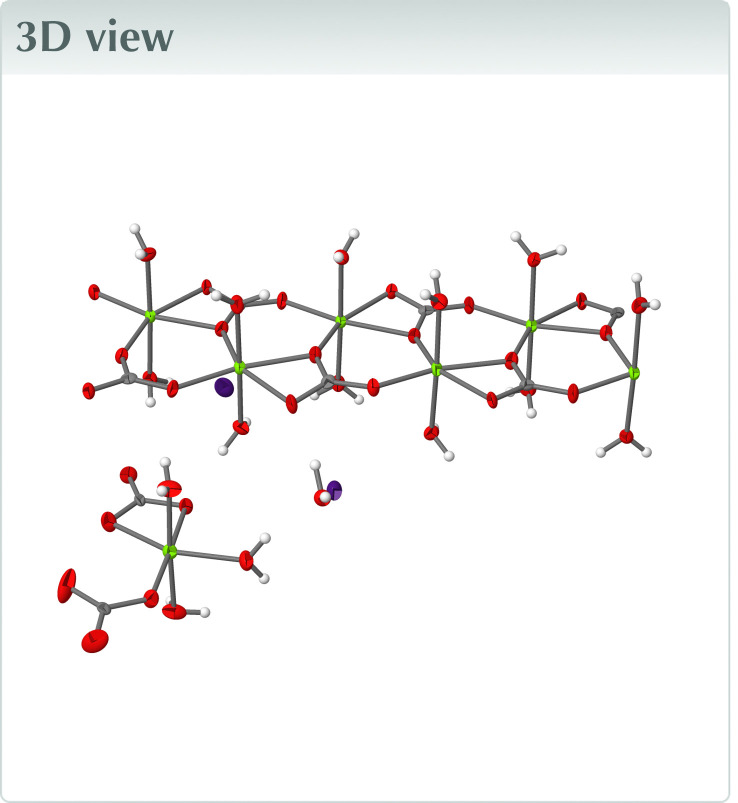

## Structure description

Up to now, only two magnesium salts containing hydrogenbiscarbonate anions [H(CO_3_)_2_^3–^] were known, *viz*. KMgH(CO_3_)_2_·4H_2_O and RbMgH(CO_3_)_2_·4H_2_O. Both can be crystallized at room temperature by combination of an aqueous solution of the alkali metal bicarbonate and an aqueous solution of magnesium chloride or nitrate (Fernandes *et al.*, 1988 ▸; Dahm, 2000 ▸). The synthesis of the analogous caesium compound was not successful (Gloss, 1937 ▸). Tkachev *et al.* (1978 ▸) reported on the synthesis of CsMgH(CO_3_)_2_·0.5H_2_O, but details about the conditions of formation were missing. In our current investigations on that matter, the title compound was found instead of a hydrogenbiscarbonate.

**Figure 1:**
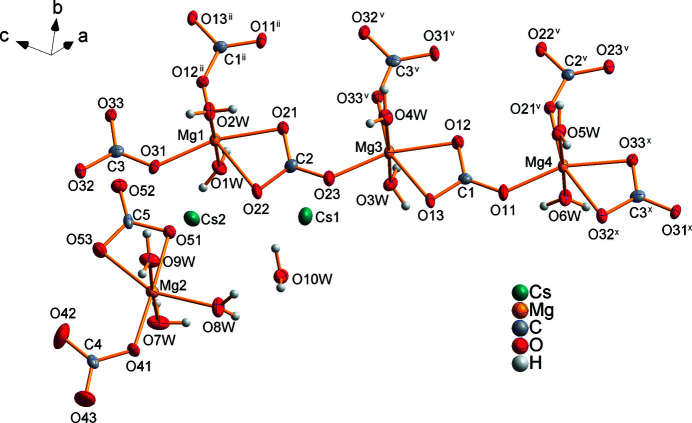
The expanded asymmetric unit of Cs_2_Mg_4_(CO_3_)_5_·10H_2_O showing the coordination polyhedra around the four Mg sites. Displacement ellipsoids are drawn at the 50% probability level. [Symmetry codes: (ii) *x* − 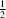 , −*y* + 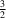 , *z* + 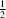 ; (v) *x* + 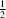 , −*y* + 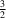 , *z* − 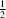 ; (*x*) *x* + 1, *y*, *z* − 1.]

**Figure 2:**
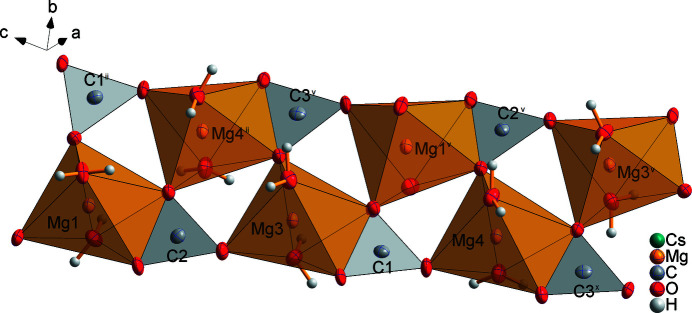
Double chain built up from octa­hedra around Mg1, Mg3 and Mg4 and carbonate units. [Symmetry codes: (ii) *x* − 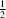 , −*y* + 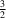 , *z* + 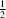 ; (v) *x* + 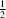 , −*y* + 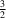 , *z* − 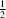 ; (*x*) *x* + 1, *y*, *z* − 1.]

**Figure 3:**
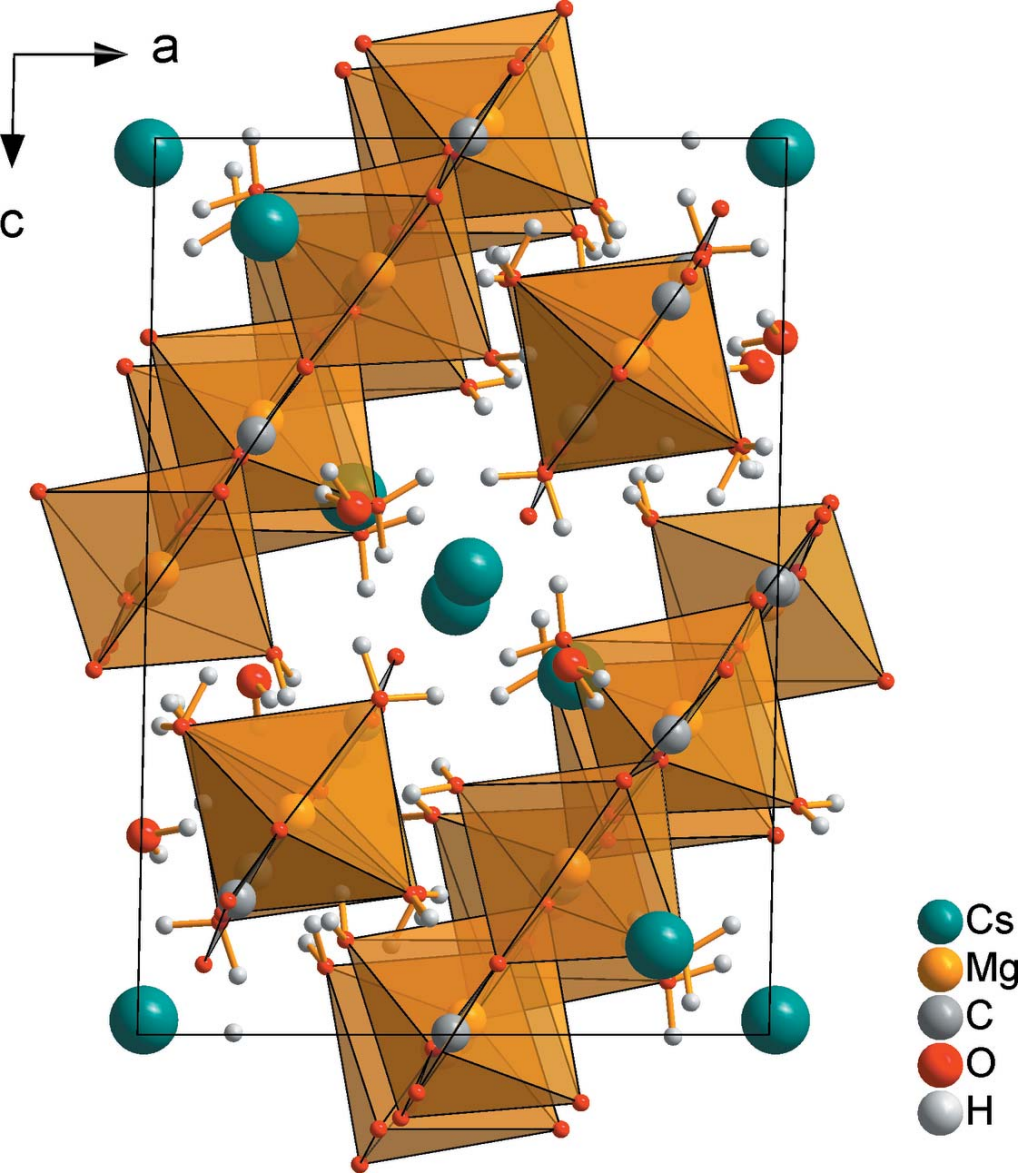
The crystal structure of Cs_2_Mg_4_(CO_3_)_5_·10H_2_O in a view along the *b* axis.

**Figure 4:**
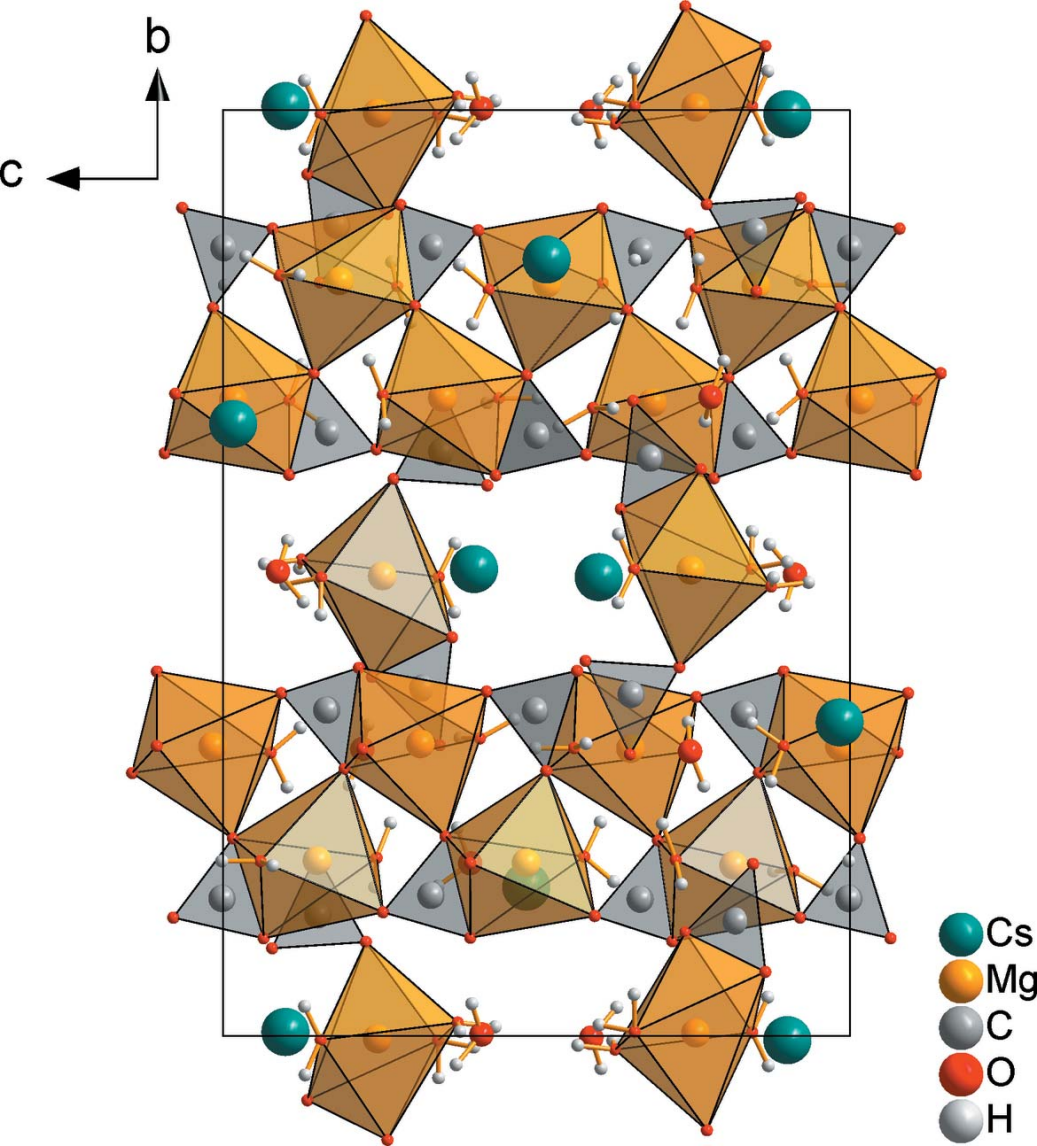
The crystal structure of Cs_2_Mg_4_(CO_3_)_5_·10H_2_O in a view along the *a* axis.

Cs_2_Mg_4_(CO_3_)_5_·10H_2_O crystallizes in the space group *P*2_1_/*n* and contains four slightly distorted [MgO_6_] octa­hedra (Fig. 1 ▸). Each of the magnesium cations Mg1, Mg3 and Mg4 forms [Mg(H_2_O)_2_(CO_3_)_3_]^4–^ units that are linked by bridging carbonate anions of the carbon atoms C1, C2 and C3 (Fig. 2 ▸). Each carbonate anion bonds in a bidentate mode to one and in a monodentate to the other cations (Figs. 1 ▸, 2 ▸). In each octa­hedron, the water mol­ecules are located in the *trans*-positions and have no bridging character. In this way, double chains of composition ^1^_∞_[Mg(H_2_O)_2/1_(CO_3_)_3/3_] are formed, extending parallel to the [ 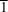 01] direction (Fig. 3 ▸). Between these double chains, isolated [Mg(H_2_O)_3_(CO_3_)_2_]^2–^ units involving the Mg2 cation are located (Fig. 4 ▸). The Mg2 cation is also octa­hedrally coordinated, in this case by three water mol­ecules and two carbonate anions (C4, C5) in a monodentate and a bidentate fashion, respectively (Fig. 1 ▸). The ^1^_∞_[Mg(H_2_O)_2/1_(CO_3_)_3/3_] double chains and the isolated [Mg(H_2_O)_3_(CO_3_)_2_]^2–^ units form alternating sheets parallel to (010) (Fig. 4 ▸).

The C—O bonds of the carbonate units range from 1.252 (12) to 1.316 (11) Å and the O—C—O angles from 114.8 (8) to 124.4 (8)°. In comparison with other crystal structures containing carbonate units and deposited in the Inorganic Structure Database (ICSD; Zagorac *et al.*, 2019 ▸), these deviations of the bond lengths and angles from ideal values of a equilateral triangle are not unusual (Cirpus, 1997 ▸). As a result, the symmetry of the carbonate anions deviates significantly from ideal *D*_3*h*_; however, the sum of all O—C—O angles remains 360° and planarity is kept, which is typical for all carbonate structures (Zemann, 1981 ▸; Cirpus, 1997 ▸).

**Figure 5:**
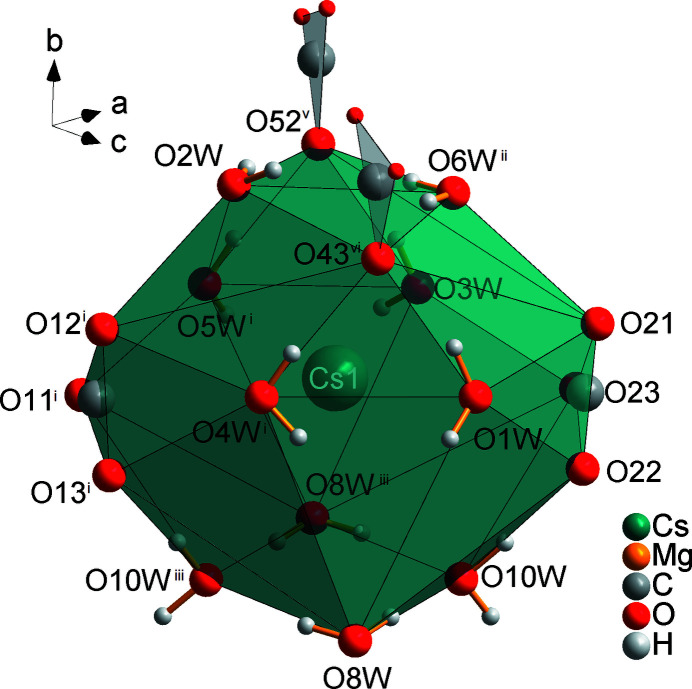
Coordination sphere of the Cs1 cation, with all atoms drawn as spheres of arbitrary radii (oxygen atoms not coordinating to Cs with half of the size of other O atoms). [Symmetry codes: (i) *x* − 1, *y*, *z*; (ii) *x* − 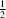 , −*y* + 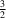 , *z* + 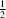 ; (iii) −*x* + 2, −*y* + 1, −*z*; (iv) *x* − 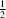 , −*y* + 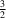 , *z* − 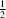 ; (v) *x* + 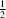 , −*y* + 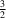 , *z* − 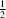 ; (vi) −*x* + 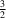 , *y* + 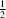 , −*z* + 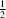 .]

**Figure 6:**
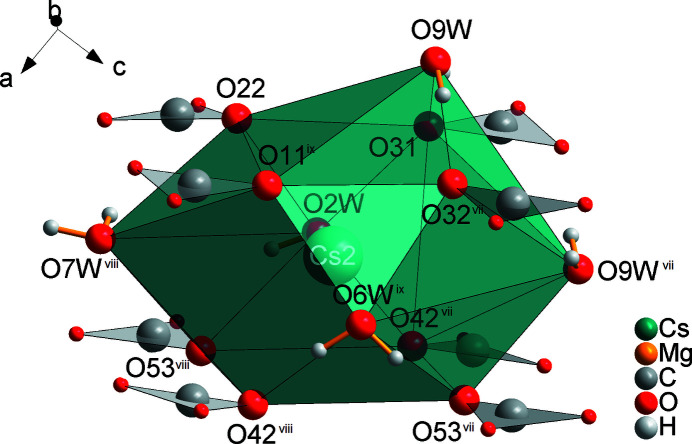
Coordination sphere of the Cs2 cation, with all atoms drawn as spheres of arbitrary radii (oxygen atoms not coordinating to Cs with half of the size of other O atoms). [Symmetry codes: (vii) −*x* + 2, −*y* + 1, −*z* + 1; (viii) *x* + 1, *y*, *z*; (ix) −*x* + 3, −*y* + 1, -*z.*]

**Figure 7:**
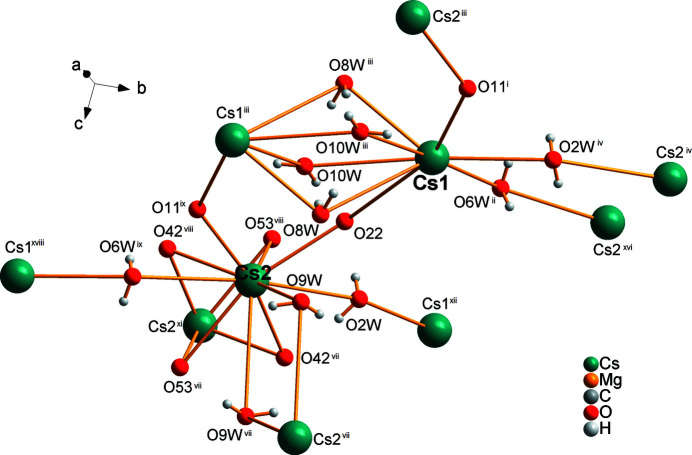
Linkage of Cs cations by O atoms, with all atoms drawn as spheres of arbitrary radii (other atoms are left out for clarity). [Symmetry codes: (i) *x* − 1, *y*, *z*; (ii) *x* − 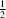 , −*y* + 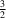 , *z* + 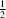 ; (iii) −*x* + 2, −*y* + 1, −*z*; (iv) *x* − 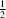 , −*y* + 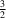 , *z* − 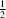 ; (vii) −*x* + 2, −*y* + 1, −*z* + 1; (viii) *x* + 1, *y*, *z*; (ix) −*x* + 3, −*y* + 1, −*z*; (xi) −*x* + 3, −*y* + 1, −*z* + 1; (xvi) −*x* + 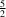 , *y* + 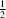 , −*z* + 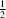 ; (xviii) −*x* + 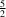 , *y* − 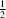 , −*z* + 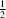 .]

The two caesium cations inter­connect two adjacent double chains and four (Cs1) and three (Cs2) [Mg2(H_2_O)_3_(CO_3_)_2_]^2–^ units, respectively, thereby generating a three-dimensional framework. The coordination numbers of the caesium cations are [6 + 12] for Cs1 and [7 + 6] for Cs2, whereby the first numeral indicates the number of the coordinating O atoms with a distance between 3.06 to 3.44 Å and the second number the number of O atoms with a distance between 3.44 and 4.12 Å. The Cs1 cation is coordinated by ten water mol­ecules and four carbonate units (Fig. 5 ▸), the Cs2 cation by five water mol­ecules and eight carbonate units (Fig. 6 ▸). The [Cs1O_18_] polyhedron is connected with another [Cs1O_18_] polyhedron by face-sharing through O8*W*, O8*W* ^iii^, O10*W* and O10*W* ^iii^ and is also linked by sharing corners to four [Cs2O_13_] polyhedra through O2*W* ^iv^, O11^i^, O6*W* ^ii^ and O22. Likewise, a [Cs2O_13_] polyhedron is linked by face-sharing through O42^vii^, O42^viii^, O53^vii^ and O53^viii^ with another [Cs2O_13_] polyhedron and by edge-sharing with two [Cs2O_13_] polyhedra through O9*W* and O9*W* ^vii^ (Fig. 7 ▸).

**Figure 8:**
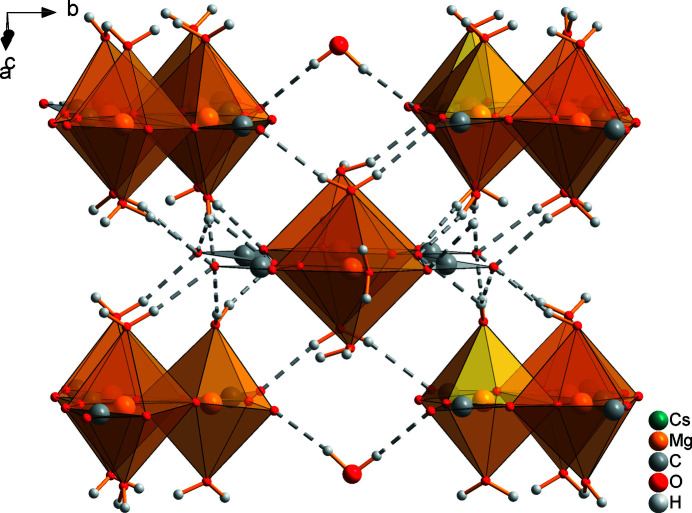
Inter­connection of double chains (at the corners), the [Mg2(H_2_O)_3_(CO_3_)_2_]^2–^ units (in the middle) and the free water mol­ecule *via* hydrogen bonds in the crystal structure of Cs_2_Mg_4_(CO_3_)_5_·10H_2_O. Hydrogen bonds are represented by dashed lines; Cs atoms are omitted for clarity.

Additional stability in the crystal structure is accomplished by hydrogen bonds between the water mol­ecules of the double chains, the [Mg2(H_2_O)_3_(CO_3_)_2_]^2–^ units and the free water mol­ecule (H25*A*—O25—H25*B*) (Fig. 8 ▸, Table 1 ▸). The shortest hydrogen bonds are built between O4*W*—H4*B*⋯O51^i^, O10*W*—H10*B*⋯O22, O4*W*—H4*A*⋯O43^ii^ and O6*W*—H6*B*⋯O52^vi^ with H⋯O distances < 2.00 Å (Table 1 ▸). The bond length correlates with the strength of the hydrogen bonds (Steiner, 2002 ▸), and in the present case the strength of the hydrogen bonding is considered to be moderate.

For the crystal structures of other alkali metal magnesium carbonates and hydrogen bis­(carbonates), see: KMgH(CO_3_)_2_·4H_2_O (Fernandes *et al.*, 1988 ▸), RbMgH(CO_3_)_2_·4H_2_O (Dahm, 2000 ▸), K_2_Mg(CO_3_)_2_·4H_2_O (Bucat *et al.*, 1977 ▸), Rb_2_Mg(CO_3_)_2_·4H_2_O (Zheng & Adam, 1994 ▸), Cs_2_Mg(CO_3_)_2_·4H_2_O (Zheng & Adam, 1999 ▸).

## Synthesis and crystallization

The synthesis was derived from the information for crystallization of KMgH(CO_3_)_2_·4H_2_O as reported by Fernandes *et al.* (1988 ▸). CO_2_ was bubbled through a solution of Cs_2_CO_3_ (9.043 g, Merck, > 99.5%) and water (22.120 g) for three h. Afterwards, a solution of Mg(NO_3_)_2_·6H_2_O (1.041 g, Merck, p.a.) and water (2.643 g) was added and stored in a sealed bottle for 2 d. A crystalline solid was formed and filtered off. The characterization with powder X-ray diffraction showed that the product was a mixture of MgCO_3_·3H_2_O and the title compound.

After filtration the remaining solution was stored in a sealed bottle for 14 d at room temperature. During this period further acicular crystals (200 *x* 20 µm) were formed. The product was washed with ethanol and characterized by powder X-ray diffraction, thermal analysis, FT–IR spectroscopy and SEM (see: supporting information). Some crystals were kept in the mother solution in a sealed vessel for one month. Afterwards a suitable crystal for the single-crystal determination was selected.

## Refinement

Crystal data, data collection and structure refinement details are summarized in Table 2 ▸. If twinning is not accounted for, the reflections can be indexed by a large ortho­rhom­bic cell, but the subsequent refinement did not result in an useful structure model. Close inspection of the diffraction pattern revealed the presence of two monoclinic cells (*P*2_1_/*n*) with lattice parameters of *a* = 9.1617 (9), *b* = 19.233 (3), *c* = 13.0065 (13) Å, *β* = 91.136 (8)°. Therefore the crystal under investigation exhibited twinning by reticular pseudomerohedry; the matrix that relates the individual diffraction pattern was determined as (− 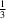 0 − 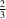 , 0 −1 0, − 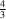 0 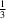 ). The reflections of both domains were integrated concurrently, leading to the following numbers. Reflections belonging to domain 1: 16785; reflections belonging to domain 2: 26839; overlaid reflections: 10073; major twin component fraction: 56%.

Structure solution permitted the assignment of all heavy atoms and the subsequent refinement leads to a chemical sensible atomic arrangement. All H atoms were discernable from difference-Fourier maps and refined with an O—H distance restraint of 0.82(2) Å and *U*_iso_(H) = 1.2*U*_eq_(O).

## Supplementary Material

Crystal structure: contains datablock(s) I. DOI: 10.1107/S2414314620001650/wm4122sup1.cif

Structure factors: contains datablock(s) I. DOI: 10.1107/S2414314620001650/wm4122Isup2.hkl

Supporting information file. DOI: 10.1107/S2414314620001650/wm4122Isup4.cml

Characterization of the title compound by thermal analysis, SEM, powder X-ray diffraction and IR spectroscopy. DOI: 10.1107/S2414314620001650/wm4122sup3.pdf

CCDC reference: 1982137

Additional supporting information:  crystallographic information; 3D view; checkCIF report

## Figures and Tables

**Table 1:** Hydrogen-bond geometry (Å, °)

*D*—H⋯*A*	*D*—H	H⋯*A*	*D*⋯*A*	*D*—H⋯*A*
O1*W*—H1*A*⋯O51	0.82 (5)	1.85 (6)	2.658 (10)	172 (12)
O1*W*—H1*B*⋯O43^i^	0.83 (5)	1.89 (6)	2.686 (11)	162 (12)
O2*W*—H2*A*⋯O52^ii^	0.80 (5)	2.01 (6)	2.783 (11)	160 (12)
O2*W*—H2*B*⋯O42^iii^	0.81 (5)	1.93 (6)	2.734 (12)	170 (13)
O3*W*—H3*A*⋯O52^iv^	0.82 (5)	2.23 (6)	2.993 (12)	157 (11)
O3*W*—H3*B*⋯O41^v^	0.82 (5)	1.92 (6)	2.737 (11)	175 (12)
O4*W*—H4*A*⋯O43^vi^	0.82 (5)	1.93 (6)	2.724 (11)	162 (13)
O4*W*—H4*B*⋯O51^ii^	0.82 (5)	1.84 (6)	2.661 (9)	174 (14)
O5*W*—H5*A*⋯O52^vii^	0.82 (5)	2.04 (6)	2.853 (10)	169 (13)
O5*W*—H5*B*⋯O41^viii^	0.80 (5)	2.05 (6)	2.831 (10)	164 (13)
O6*W*—H6*A*⋯O43^v^	0.84 (5)	2.21 (6)	3.021 (13)	165 (13)
O6*W*—H6*B*⋯O52^ix^	0.81 (5)	1.98 (6)	2.783 (10)	174 (13)
O7*W*—H7*B*⋯O23^x^	0.83 (5)	2.07 (7)	2.843 (10)	155 (13)
O8*W*—H8*A*⋯O10*W*	0.82 (5)	2.02 (6)	2.815 (10)	165 (13)
O8*W*—H8*B*⋯O10*W* ^v^	0.82 (5)	2.02 (7)	2.815 (10)	164 (15)
O9*W*—H9*A*⋯O31	0.82 (5)	2.09 (7)	2.869 (10)	161 (13)
O9*W*—H9*B*⋯O32^iii^	0.82 (5)	1.87 (7)	2.673 (10)	170 (13)
O10*W*—H10*A*⋯O13^viii^	0.81 (5)	2.01 (6)	2.798 (9)	162 (13)
O10*W*—H10*B*⋯O22	0.90 (13)	1.84 (13)	2.730 (10)	169 (12)

Symmetry codes: (i) 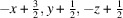 ; (ii) 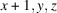 ; (iii) 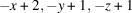 ; (iv) 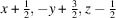 ; (v) 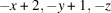 ; (vi) 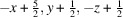 ; (vii) 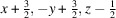 ; (viii) 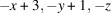 ; (ix) 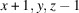 ; (x) 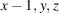 .

**Table 2:** Experimental details

Crystal data	
Chemical formula	Cs_2_Mg_4_(CO_3_)_5_·10H_2_O
*M*_r_	843.27
Crystal system, space group	Monoclinic, *P*2_1_/*n*
Temperature (K)	200
*a*, *b*, *c* (Å)	9.1617 (9), 19.233 (3), 13.0065 (13)
β (°)	91.136 (8)
*V* (Å^3^)	2291.4 (4)
*Z*	4
Radiation type	Mo *K*α
μ (mm^−1^)	3.41
Crystal size (mm)	0.6 × 0.45 × 0.25

Data collection	
Diffractometer	STOE *IPDS* 2T
Absorption correction	Integration (Coppens, 1970 ▸)
*T*_min_, *T*_max_	0.151, 0.439
No. of measured, independent and observed [*I* > 2σ(*I*)] reflections	37526, 37526, 31786
*R*_int_	0.086
(sin θ/λ)_max_ (Å^−1^)	0.650

Refinement	
*R*[*F* ^2^ > 2σ(*F* ^2^)], *wR*(*F* ^2^), *S*	0.073, 0.274, 1.22
No. of reflections	37526
No. of parameters	386
No. of restraints	19
H-atom treatment	Only H-atom coordinates refined
Δρ_max_, Δρ_min_ (e Å^−3^)	3.48, −2.86

Computer programs: *X-AREA* and *X-RED* (Stoe & Cie, 2015 ▸), *SHELXS97* (Sheldrick, 2008 ▸), *SHELXL2016/6* (Sheldrick, 2015 ▸), *DIAMOND* (Brandenburg, 2017 ▸) and *publCIF* (Westrip, 2010 ▸).

## full crystallographic data

### Crystal data

**Table:**

Cs_2_Mg_4_(CO_3_)_5_·10H_2_O	*F*(000) = 1632
*M**_r_* = 843.27	*D*_x_ = 2.444 Mg m^−^^3^
Monoclinic, *P*2_1_/*n*	Mo *K*α radiation, λ = 0.71073 Å
*a* = 9.1617 (9) Å	Cell parameters from 11266 reflections
*b* = 19.233 (3) Å	θ = 10.0–27.5°
*c* = 13.0065 (13) Å	µ = 3.41 mm^−^^1^
β = 91.136 (8)°	*T* = 200 K
*V* = 2291.4 (4) Å^3^	Needle, colorless
*Z* = 4	0.6 × 0.45 × 0.25 mm

### Data collection

**Table:**

STOE IPDS 2T diffractometer	37526 independent reflections
Radiation source: sealed X-ray tube, 12 x 0.4 mm long-fine focus	31786 reflections with *I* > 2σ(*I*)
Plane graphite monochromator	*R*_int_ = 0.086
Detector resolution: 6.67 pixels mm^-1^	θ_max_ = 27.5°, θ_min_ = 2.7°
rotation method scans	*h* = −11→11
Absorption correction: integration (Coppens, 1970)	*k* = −24→24
*T*_min_ = 0.151, *T*_max_ = 0.439	*l* = −16→16
37526 measured reflections	

### Refinement

**Table:**

Refinement on *F*^2^	19 restraints
Least-squares matrix: full	Hydrogen site location: difference Fourier map
*R*[*F*^2^ > 2σ(*F*^2^)] = 0.073	Only H-atom coordinates refined
*wR*(*F*^2^) = 0.274	*w* = 1/[σ^2^(*F*_o_^2^) + (0.2*P*)^2^] where *P* = (*F*_o_^2^ + 2*F*_c_^2^)/3
*S* = 1.22	(Δ/σ)_max_ = 0.001
37526 reflections	Δρ_max_ = 3.48 e Å^−^^3^
386 parameters	Δρ_min_ = −2.86 e Å^−^^3^

### Special details

**Table:**

Geometry. All esds (except the esd in the dihedral angle between two l.s. planes) are estimated using the full covariance matrix. The cell esds are taken into account individually in the estimation of esds in distances, angles and torsion angles; correlations between esds in cell parameters are only used when they are defined by crystal symmetry. An approximate (isotropic) treatment of cell esds is used for estimating esds involving l.s. planes.
Refinement. Refined as a two-component twin. All H atoms were discernable from difference Fourier maps. Their *U*_iso_ values were set at 1.2*U*_eq_(O) using a riding-model approximation, with O—H distance restraint of 0.82 (2) Å.

### Fractional atomic coordinates and isotropic or equivalent isotropic displacement parameters (Å^2^)

**Table:**

	*x*	*y*	*z*	*U*_iso_*/*U*_eq_	
Cs1	1.00975 (7)	0.66121 (4)	−0.01626 (5)	0.0303 (3)	
Cs2	1.32369 (7)	0.49560 (3)	0.40043 (5)	0.0264 (3)	
Mg1	1.1797 (3)	0.68437 (15)	0.3146 (2)	0.0151 (6)	
Mg2	0.7423 (3)	0.50315 (14)	0.2539 (2)	0.0180 (6)	
Mg3	1.5193 (3)	0.68707 (16)	−0.0165 (2)	0.0149 (6)	
Mg4	1.8523 (3)	0.68950 (15)	−0.3500 (2)	0.0148 (6)	
C1	1.6693 (10)	0.6520 (4)	−0.1682 (7)	0.0151 (17)	
C2	1.3367 (10)	0.6479 (5)	0.1655 (7)	0.0157 (17)	
C3	0.9960 (9)	0.6486 (5)	0.4985 (7)	0.0159 (18)	
C4	0.6528 (9)	0.3691 (5)	0.3489 (7)	0.0159 (15)	
C5	0.6780 (9)	0.6242 (5)	0.3186 (6)	0.0161 (15)	
O1W	0.9961 (8)	0.6901 (4)	0.2222 (6)	0.0214 (13)	
H1A	0.927 (10)	0.664 (6)	0.231 (10)	0.026*	
H1B	0.972 (13)	0.729 (4)	0.201 (10)	0.026*	
O2W	1.3628 (8)	0.6785 (4)	0.4098 (6)	0.0226 (14)	
H2A	1.437 (9)	0.678 (7)	0.378 (8)	0.027*	
H2B	1.375 (14)	0.658 (6)	0.464 (6)	0.027*	
O31	1.0684 (8)	0.6279 (4)	0.4208 (5)	0.0200 (13)	
O21	1.3187 (7)	0.7114 (3)	0.1938 (5)	0.0185 (12)	
O22	1.2623 (8)	0.6024 (3)	0.2168 (5)	0.0211 (13)	
O23	1.4196 (8)	0.6297 (4)	0.0929 (5)	0.0197 (13)	
O3W	1.3266 (8)	0.6867 (4)	−0.1057 (6)	0.0204 (13)	
H3B	1.318 (14)	0.662 (6)	−0.157 (7)	0.025*	
H3A	1.301 (13)	0.725 (4)	−0.126 (9)	0.025*	
O4W	1.7042 (8)	0.6938 (4)	0.0761 (6)	0.0220 (14)	
H4B	1.726 (14)	0.667 (6)	0.123 (8)	0.026*	
H4A	1.734 (14)	0.731 (4)	0.102 (9)	0.026*	
O5W	2.0403 (8)	0.6925 (4)	−0.2549 (6)	0.0218 (14)	
H5B	2.097 (11)	0.661 (5)	−0.258 (10)	0.026*	
H5A	2.074 (13)	0.730 (4)	−0.233 (10)	0.026*	
O6W	1.6622 (8)	0.6890 (4)	−0.4404 (6)	0.0227 (14)	
H6A	1.576 (7)	0.684 (7)	−0.421 (10)	0.027*	
H6B	1.652 (14)	0.690 (7)	−0.502 (4)	0.027*	
O11	1.7447 (8)	0.6338 (4)	−0.2448 (5)	0.0220 (14)	
O12	1.6439 (8)	0.7159 (3)	−0.1449 (5)	0.0200 (13)	
O13	1.6152 (7)	0.6063 (3)	−0.1054 (5)	0.0176 (12)	
O7W	0.5600 (9)	0.5047 (3)	0.1568 (7)	0.0302 (16)	
H7A	0.601 (15)	0.512 (8)	0.100 (7)	0.036*	
H7B	0.527 (14)	0.545 (4)	0.156 (11)	0.036*	
O8W	0.8731 (8)	0.4854 (4)	0.1256 (5)	0.0280 (14)	
H8A	0.960 (7)	0.495 (6)	0.122 (12)	0.034*	
H8B	0.843 (15)	0.490 (7)	0.066 (5)	0.034*	
O51	0.7580 (7)	0.6121 (3)	0.2376 (5)	0.0182 (12)	
O52	0.6504 (8)	0.6860 (3)	0.3457 (6)	0.0231 (13)	
O41	0.7184 (8)	0.3984 (3)	0.2720 (5)	0.0241 (13)	
O42	0.6027 (12)	0.4052 (5)	0.4216 (7)	0.049 (2)	
O43	0.6392 (11)	0.3043 (4)	0.3508 (7)	0.040 (2)	
O32	0.9441 (7)	0.6056 (3)	0.5649 (5)	0.0186 (13)	
O33	0.9686 (8)	0.7136 (3)	0.5157 (5)	0.0177 (12)	
O9W	0.9293 (9)	0.5041 (3)	0.3441 (6)	0.0283 (15)	
H9B	0.959 (14)	0.468 (4)	0.370 (10)	0.034*	
H9A	0.949 (14)	0.542 (4)	0.369 (10)	0.034*	
O53	0.6312 (7)	0.5693 (4)	0.3652 (5)	0.0261 (14)	
O10W	1.1733 (8)	0.4982 (3)	0.0876 (5)	0.0222 (14)	
H10A	1.223 (12)	0.465 (5)	0.103 (9)	0.027*	
H10B	1.214 (14)	0.531 (7)	0.128 (10)	0.027*	

### Atomic displacement parameters (Å^2^)

**Table:**

	*U*^11^	*U*^22^	*U*^33^	*U*^12^	*U*^13^	*U*^23^
Cs1	0.0293 (4)	0.0358 (4)	0.0260 (4)	−0.0016 (2)	0.0086 (3)	−0.0031 (2)
Cs2	0.0259 (4)	0.0232 (4)	0.0301 (4)	−0.00246 (19)	0.0027 (3)	0.0020 (2)
Mg1	0.0138 (15)	0.0184 (14)	0.0132 (15)	−0.0001 (10)	0.0024 (11)	−0.0003 (11)
Mg2	0.0192 (15)	0.0154 (14)	0.0195 (14)	−0.0015 (9)	0.0023 (12)	0.0001 (10)
Mg3	0.0136 (14)	0.0178 (14)	0.0135 (15)	0.0011 (10)	0.0036 (11)	0.0000 (10)
Mg4	0.0127 (14)	0.0188 (14)	0.0129 (15)	0.0006 (10)	0.0027 (11)	0.0000 (11)
C1	0.015 (4)	0.015 (4)	0.015 (4)	−0.002 (3)	−0.004 (3)	0.000 (3)
C2	0.016 (4)	0.018 (4)	0.013 (4)	0.003 (3)	−0.003 (3)	0.001 (3)
C3	0.014 (4)	0.018 (4)	0.015 (4)	0.001 (3)	−0.008 (3)	0.000 (3)
C4	0.010 (3)	0.023 (4)	0.015 (4)	−0.004 (3)	−0.002 (3)	0.003 (3)
C5	0.010 (4)	0.027 (4)	0.011 (4)	−0.004 (3)	0.000 (3)	0.002 (3)
O1W	0.016 (3)	0.023 (3)	0.025 (4)	−0.003 (2)	−0.001 (3)	0.003 (3)
O2W	0.017 (3)	0.035 (4)	0.016 (3)	0.002 (3)	0.002 (2)	0.006 (3)
O31	0.023 (3)	0.022 (3)	0.015 (3)	0.001 (2)	0.008 (3)	−0.004 (2)
O21	0.022 (3)	0.017 (3)	0.017 (3)	0.001 (2)	0.006 (2)	0.001 (2)
O22	0.021 (3)	0.022 (3)	0.021 (3)	−0.001 (2)	0.011 (3)	0.003 (2)
O23	0.019 (3)	0.022 (3)	0.018 (3)	0.002 (2)	0.007 (3)	0.001 (2)
O3W	0.019 (3)	0.023 (3)	0.019 (4)	0.002 (2)	0.000 (3)	−0.005 (3)
O4W	0.021 (3)	0.027 (3)	0.018 (3)	−0.001 (2)	−0.004 (3)	0.006 (3)
O5W	0.019 (3)	0.028 (3)	0.018 (3)	0.004 (2)	−0.001 (3)	−0.003 (3)
O6W	0.014 (3)	0.037 (4)	0.017 (3)	−0.004 (3)	0.000 (2)	−0.002 (3)
O11	0.025 (4)	0.026 (3)	0.016 (3)	0.000 (3)	0.010 (3)	0.002 (3)
O12	0.026 (4)	0.016 (3)	0.017 (3)	0.002 (2)	0.005 (3)	0.002 (2)
O13	0.015 (3)	0.022 (3)	0.016 (3)	−0.002 (2)	0.007 (2)	0.002 (2)
O7W	0.023 (4)	0.020 (3)	0.046 (4)	0.002 (2)	−0.011 (3)	−0.002 (3)
O8W	0.021 (3)	0.042 (4)	0.021 (3)	0.000 (3)	0.006 (3)	0.002 (3)
O51	0.020 (3)	0.016 (3)	0.019 (3)	0.001 (2)	0.004 (2)	0.001 (2)
O52	0.026 (3)	0.018 (3)	0.026 (3)	0.002 (2)	0.001 (3)	−0.001 (3)
O41	0.035 (3)	0.018 (3)	0.019 (3)	−0.008 (3)	0.007 (3)	0.002 (2)
O42	0.075 (6)	0.044 (5)	0.030 (4)	0.027 (4)	0.023 (4)	0.000 (3)
O43	0.070 (6)	0.021 (3)	0.030 (4)	−0.009 (4)	−0.007 (4)	0.009 (3)
O32	0.018 (3)	0.022 (3)	0.016 (3)	−0.001 (2)	0.005 (2)	0.002 (2)
O33	0.021 (3)	0.017 (3)	0.015 (3)	0.001 (2)	0.004 (2)	−0.001 (2)
O9W	0.030 (4)	0.019 (3)	0.035 (4)	0.000 (2)	−0.012 (3)	0.004 (3)
O53	0.030 (3)	0.019 (3)	0.029 (3)	−0.001 (2)	0.010 (3)	0.003 (3)
O10W	0.020 (3)	0.024 (4)	0.023 (3)	0.000 (2)	0.001 (3)	−0.002 (2)

### Geometric parameters (Å, º)

**Table:**

Cs1—O4W^i^	3.132 (8)	Mg1—O21	2.107 (8)
Cs1—O1W	3.156 (8)	Mg1—O22	2.171 (8)
Cs1—O5W^i^	3.180 (8)	Mg2—O41	2.041 (7)
Cs1—O3W	3.187 (8)	Mg2—O9W	2.057 (9)
Cs1—O6W^ii^	3.342 (8)	Mg2—O7W	2.074 (9)
Cs1—O8W^iii^	3.344 (8)	Mg2—O8W	2.102 (7)
Cs1—O2W^iv^	3.492 (8)	Mg2—O51	2.111 (7)
Cs1—O10W^iii^	3.607 (7)	Mg2—O53	2.193 (7)
Cs1—O52^v^	3.689 (7)	Mg3—O33^v^	2.012 (8)
Cs1—O10W	3.718 (7)	Mg3—O23	2.032 (7)
Cs1—O43^vi^	3.767 (8)	Mg3—O4W	2.063 (7)
Cs1—O11^i^	3.837 (7)	Mg3—O3W	2.093 (7)
Cs1—O12^i^	3.862 (7)	Mg3—O12	2.116 (8)
Cs1—O13^i^	3.920 (7)	Mg3—O13	2.137 (7)
Cs1—O22	3.943 (7)	Mg4—O11	2.011 (7)
Cs1—O21	4.013 (7)	Mg4—O21^v^	2.015 (7)
Cs1—O23	4.034 (7)	Mg4—O6W	2.082 (7)
Cs1—O8W	4.062 (8)	Mg4—O5W	2.101 (7)
Cs2—O42^vii^	3.065 (10)	Mg4—O33^x^	2.115 (8)
Cs2—O42^viii^	3.099 (9)	Mg4—O32^x^	2.139 (7)
Cs2—O32^vii^	3.171 (7)	C1—O11	1.272 (11)
Cs2—O22	3.191 (7)	C1—O12	1.289 (11)
Cs2—O53^viii^	3.195 (7)	C1—O13	1.305 (11)
Cs2—O11^ix^	3.261 (7)	C2—O23	1.272 (11)
Cs2—O53^vii^	3.312 (7)	C2—O21	1.286 (11)
Cs2—O31	3.470 (7)	C2—O22	1.302 (11)
Cs2—O2W	3.537 (8)	C3—O31	1.283 (11)
Cs2—O6W^ix^	3.590 (8)	C3—O32	1.292 (11)
Cs2—O9W	3.676 (8)	C3—O33	1.296 (12)
Cs2—O7W^viii^	3.877 (9)	C4—O43	1.252 (12)
Cs2—O9W^vii^	4.090 (9)	C4—O42	1.267 (12)
Mg1—O12^ii^	2.017 (7)	C4—O41	1.305 (11)
Mg1—O31	2.045 (8)	C5—O52	1.266 (12)
Mg1—O1W	2.050 (8)	C5—O53	1.296 (11)
Mg1—O2W	2.068 (7)	C5—O51	1.316 (11)
			
O4W^i^—Cs1—O1W	62.25 (18)	O11^ix^—Cs2—O9W^vii^	113.60 (15)
O4W^i^—Cs1—O5W^i^	115.68 (19)	O53^vii^—Cs2—O9W^vii^	46.77 (15)
O1W—Cs1—O5W^i^	158.76 (19)	O31—Cs2—O9W^vii^	62.82 (15)
O4W^i^—Cs1—O3W	159.55 (19)	O2W—Cs2—O9W^vii^	91.69 (16)
O1W—Cs1—O3W	112.70 (19)	O6W^ix^—Cs2—O9W^vii^	84.43 (16)
O5W^i^—Cs1—O3W	61.06 (18)	O9W—Cs2—O9W^vii^	65.87 (19)
O4W^i^—Cs1—O6W^ii^	94.90 (19)	O7W^viii^—Cs2—O9W^vii^	177.29 (14)
O1W—Cs1—O6W^ii^	65.32 (18)	O12^ii^—Mg1—O31	104.0 (3)
O5W^i^—Cs1—O6W^ii^	94.64 (19)	O12^ii^—Mg1—O1W	88.1 (3)
O3W—Cs1—O6W^ii^	66.23 (17)	O31—Mg1—O1W	90.6 (3)
O4W^i^—Cs1—O8W^iii^	128.97 (18)	O12^ii^—Mg1—O2W	91.7 (3)
O1W—Cs1—O8W^iii^	125.86 (18)	O31—Mg1—O2W	88.6 (3)
O5W^i^—Cs1—O8W^iii^	73.09 (18)	O1W—Mg1—O2W	179.1 (3)
O3W—Cs1—O8W^iii^	70.86 (17)	O12^ii^—Mg1—O21	93.7 (3)
O6W^ii^—Cs1—O8W^iii^	135.78 (18)	O31—Mg1—O21	162.0 (3)
O4W^i^—Cs1—O2W^iv^	65.55 (17)	O1W—Mg1—O21	92.9 (3)
O1W—Cs1—O2W^iv^	95.32 (18)	O2W—Mg1—O21	88.0 (3)
O5W^i^—Cs1—O2W^iv^	66.73 (17)	O12^ii^—Mg1—O22	154.6 (3)
O3W—Cs1—O2W^iv^	96.47 (18)	O31—Mg1—O22	101.2 (3)
O6W^ii^—Cs1—O2W^iv^	58.45 (17)	O1W—Mg1—O22	89.3 (3)
O8W^iii^—Cs1—O2W^iv^	138.81 (18)	O2W—Mg1—O22	91.3 (3)
O4W^i^—Cs1—O10W^iii^	81.63 (18)	O21—Mg1—O22	61.3 (3)
O1W—Cs1—O10W^iii^	112.04 (17)	O41—Mg2—O9W	91.9 (3)
O5W^i^—Cs1—O10W^iii^	87.65 (17)	O41—Mg2—O7W	89.9 (3)
O3W—Cs1—O10W^iii^	117.33 (17)	O9W—Mg2—O7W	176.9 (4)
O6W^ii^—Cs1—O10W^iii^	176.43 (18)	O41—Mg2—O8W	89.7 (3)
O8W^iii^—Cs1—O10W^iii^	47.59 (17)	O9W—Mg2—O8W	88.4 (3)
O2W^iv^—Cs1—O10W^iii^	120.32 (16)	O7W—Mg2—O8W	89.1 (3)
O4W^i^—Cs1—O52^v^	110.53 (17)	O41—Mg2—O51	177.6 (3)
O1W—Cs1—O52^v^	111.04 (17)	O9W—Mg2—O51	89.5 (3)
O5W^i^—Cs1—O52^v^	48.39 (17)	O7W—Mg2—O51	88.9 (3)
O3W—Cs1—O52^v^	50.97 (17)	O8W—Mg2—O51	92.3 (3)
O6W^ii^—Cs1—O52^v^	46.30 (17)	O41—Mg2—O53	116.4 (3)
O8W^iii^—Cs1—O52^v^	110.27 (17)	O9W—Mg2—O53	90.6 (3)
O2W^iv^—Cs1—O52^v^	45.51 (17)	O7W—Mg2—O53	90.9 (3)
O10W^iii^—Cs1—O52^v^	135.79 (15)	O8W—Mg2—O53	153.9 (3)
O4W^i^—Cs1—O10W	112.73 (17)	O51—Mg2—O53	61.6 (3)
O1W—Cs1—O10W	79.33 (17)	O33^v^—Mg3—O23	105.2 (3)
O5W^i^—Cs1—O10W	118.15 (17)	O33^v^—Mg3—O4W	90.5 (3)
O3W—Cs1—O10W	84.19 (17)	O23—Mg3—O4W	90.0 (3)
O6W^ii^—Cs1—O10W	117.17 (16)	O33^v^—Mg3—O3W	85.6 (3)
O8W^iii^—Cs1—O10W	46.62 (16)	O23—Mg3—O3W	90.0 (3)
O2W^iv^—Cs1—O10W	174.40 (16)	O4W—Mg3—O3W	176.0 (3)
O10W^iii^—Cs1—O10W	63.82 (19)	O33^v^—Mg3—O12	92.6 (3)
O52^v^—Cs1—O10W	134.94 (15)	O23—Mg3—O12	162.2 (3)
O4W^i^—Cs1—O43^vi^	45.4 (2)	O4W—Mg3—O12	89.7 (3)
O1W—Cs1—O43^vi^	44.59 (18)	O3W—Mg3—O12	91.5 (3)
O5W^i^—Cs1—O43^vi^	117.36 (19)	O33^v^—Mg3—O13	154.5 (3)
O3W—Cs1—O43^vi^	116.1 (2)	O23—Mg3—O13	100.3 (3)
O6W^ii^—Cs1—O43^vi^	49.9 (2)	O4W—Mg3—O13	91.2 (3)
O8W^iii^—Cs1—O43^vi^	169.12 (19)	O3W—Mg3—O13	92.8 (3)
O2W^iv^—Cs1—O43^vi^	50.96 (18)	O12—Mg3—O13	61.9 (3)
O10W^iii^—Cs1—O43^vi^	126.59 (19)	O11—Mg4—O21^v^	103.4 (3)
O52^v^—Cs1—O43^vi^	80.2 (2)	O11—Mg4—O6W	88.1 (3)
O10W—Cs1—O43^vi^	123.82 (17)	O21^v^—Mg4—O6W	91.9 (3)
O4W^i^—Cs1—O11^i^	76.59 (17)	O11—Mg4—O5W	91.2 (3)
O1W—Cs1—O11^i^	138.48 (17)	O21^v^—Mg4—O5W	86.3 (3)
O5W^i^—Cs1—O11^i^	48.42 (16)	O6W—Mg4—O5W	177.9 (3)
O3W—Cs1—O11^i^	107.78 (16)	O11—Mg4—O33^x^	160.1 (3)
O6W^ii^—Cs1—O11^i^	126.97 (16)	O21^v^—Mg4—O33^x^	96.2 (3)
O8W^iii^—Cs1—O11^i^	76.01 (17)	O6W—Mg4—O33^x^	88.0 (3)
O2W^iv^—Cs1—O11^i^	70.93 (16)	O5W—Mg4—O33^x^	93.4 (3)
O10W^iii^—Cs1—O11^i^	53.13 (15)	O11—Mg4—O32^x^	98.8 (3)
C1^i^—Cs1—O11^i^	19.35 (18)	O21^v^—Mg4—O32^x^	157.5 (3)
O52^v^—Cs1—O11^i^	87.38 (15)	O6W—Mg4—O32^x^	92.2 (3)
O10W—Cs1—O11^i^	114.21 (15)	O5W—Mg4—O32^x^	89.9 (3)
O43^vi^—Cs1—O11^i^	108.19 (17)	O33^x^—Mg4—O32^x^	61.9 (3)
C2—Cs1—O11^i^	162.70 (18)	O11—C1—O12	123.4 (9)
O4W^i^—Cs1—O12^i^	48.47 (17)	O11—C1—O13	121.6 (8)
O1W—Cs1—O12^i^	109.07 (17)	O12—C1—O13	115.0 (8)
O5W^i^—Cs1—O12^i^	67.54 (17)	O23—C2—O21	123.8 (9)
O3W—Cs1—O12^i^	126.08 (17)	O23—C2—O22	121.4 (8)
O6W^ii^—Cs1—O12^i^	104.30 (15)	O21—C2—O22	114.8 (8)
O8W^iii^—Cs1—O12^i^	109.18 (16)	O31—C3—O32	122.1 (8)
O2W^iv^—Cs1—O12^i^	46.65 (15)	O31—C3—O33	122.7 (9)
O10W^iii^—Cs1—O12^i^	74.03 (15)	O32—C3—O33	115.3 (8)
O52^v^—Cs1—O12^i^	83.15 (15)	O43—C4—O42	119.5 (9)
O10W—Cs1—O12^i^	136.66 (15)	O43—C4—O41	119.5 (9)
O43^vi^—Cs1—O12^i^	74.18 (17)	O42—C4—O41	121.0 (9)
C2—Cs1—O12^i^	163.24 (17)	O43—C4—Cs2^i^	122.3 (7)
O11^i^—Cs1—O12^i^	34.05 (15)	O52—C5—O53	124.4 (8)
O4W^i^—Cs1—O13^i^	48.80 (16)	O52—C5—O51	120.5 (8)
O1W—Cs1—O13^i^	106.49 (16)	O53—C5—O51	115.1 (8)
O5W^i^—Cs1—O13^i^	82.00 (16)	Mg1—O1W—Cs1	121.2 (3)
O3W—Cs1—O13^i^	140.80 (16)	Mg1—O2W—Cs1^xi^	114.8 (3)
O6W^ii^—Cs1—O13^i^	134.21 (16)	Mg1—O2W—Cs2	87.3 (3)
O8W^iii^—Cs1—O13^i^	87.06 (16)	Cs1^xi^—O2W—Cs2	157.8 (2)
O2W^iv^—Cs1—O13^i^	78.95 (15)	Mg1—O2W—Cs2^xii^	141.2 (3)
O10W^iii^—Cs1—O13^i^	43.39 (15)	Cs1^xi^—O2W—Cs2^xii^	103.85 (15)
O52^v^—Cs1—O13^i^	113.57 (14)	Cs2—O2W—Cs2^xii^	53.93 (10)
O10W—Cs1—O13^i^	104.06 (14)	C3—O31—Mg1	129.8 (6)
O43^vi^—Cs1—O13^i^	91.34 (18)	C3—O31—Cs2	130.3 (6)
O11^i^—Cs1—O13^i^	33.70 (13)	Mg1—O31—Cs2	89.5 (2)
O12^i^—Cs1—O13^i^	32.64 (14)	C3—O31—Cs2^vii^	52.0 (5)
O4W^i^—Cs1—O22	106.13 (16)	Mg1—O31—Cs2^vii^	162.3 (3)
O1W—Cs1—O22	47.85 (16)	Cs2—O31—Cs2^vii^	100.45 (15)
O5W^i^—Cs1—O22	138.01 (16)	C2—O21—Mg4^ii^	142.7 (7)
O3W—Cs1—O22	78.46 (16)	C2—O21—Mg1	93.6 (6)
O6W^ii^—Cs1—O22	77.57 (15)	Mg4^ii^—O21—Mg1	122.8 (4)
O8W^iii^—Cs1—O22	84.08 (16)	C2—O21—Cs1	70.7 (5)
O2W^iv^—Cs1—O22	133.02 (15)	Mg4^ii^—O21—Cs1	98.3 (2)
O10W^iii^—Cs1—O22	102.53 (15)	Mg1—O21—Cs1	91.1 (2)
O52^v^—Cs1—O22	113.41 (14)	C2—O21—Cs1^xi^	131.5 (5)
O10W—Cs1—O22	41.62 (14)	Mg4^ii^—O21—Cs1^xi^	71.6 (2)
O43^vi^—Cs1—O22	89.08 (16)	Mg1—O21—Cs1^xi^	75.9 (2)
O11^i^—Cs1—O22	155.36 (15)	Cs1—O21—Cs1^xi^	154.06 (17)
O12^i^—Cs1—O22	154.45 (14)	C2—O21—Cs2	50.1 (5)
O13^i^—Cs1—O22	132.37 (15)	Mg4^ii^—O21—Cs2	161.5 (3)
O4W^i^—Cs1—O21	108.32 (16)	Mg1—O21—Cs2	52.20 (17)
O1W—Cs1—O21	47.90 (16)	Cs1—O21—Cs2	99.62 (13)
O5W^i^—Cs1—O21	123.01 (17)	Cs1^xi^—O21—Cs2	90.13 (11)
O3W—Cs1—O21	64.93 (16)	C2—O22—Mg1	90.2 (5)
O6W^ii^—Cs1—O21	46.11 (15)	C2—O22—Cs2	136.9 (5)
O8W^iii^—Cs1—O21	105.33 (16)	Mg1—O22—Cs2	95.0 (2)
O2W^iv^—Cs1—O21	103.78 (15)	C2—O22—Cs1	73.7 (5)
O10W^iii^—Cs1—O21	134.24 (15)	Mg1—O22—Cs1	92.1 (2)
O52^v^—Cs1—O21	83.76 (14)	Cs2—O22—Cs1	148.4 (2)
O10W—Cs1—O21	71.43 (14)	C2—O23—Mg3	130.8 (6)
O43^vi^—Cs1—O21	72.18 (17)	C2—O23—Cs1	69.8 (5)
O11^i^—Cs1—O21	170.94 (14)	Mg3—O23—Cs1	95.7 (2)
O12^i^—Cs1—O21	145.47 (16)	C2—O23—Cs2	54.2 (5)
O13^i^—Cs1—O21	154.19 (14)	Mg3—O23—Cs2	163.1 (3)
O22—Cs1—O21	31.80 (13)	Cs1—O23—Cs2	100.81 (14)
O4W^i^—Cs1—O23	136.71 (16)	Mg3—O3W—Cs1	124.2 (3)
O1W—Cs1—O23	74.65 (16)	Mg3—O4W—Cs1^viii^	119.6 (3)
O5W^i^—Cs1—O23	105.83 (16)	Mg4—O5W—Cs1^viii^	118.8 (3)
O3W—Cs1—O23	45.84 (16)	Mg4—O5W—Cs2^ix^	42.37 (17)
O6W^ii^—Cs1—O23	69.21 (16)	Cs1^viii^—O5W—Cs2^ix^	98.86 (18)
O8W^iii^—Cs1—O23	73.66 (16)	Mg4—O6W—Cs1^v^	120.2 (3)
O2W^iv^—Cs1—O23	125.51 (15)	Mg4—O6W—Cs2^ix^	84.0 (2)
O10W^iii^—Cs1—O23	112.81 (15)	Cs1^v^—O6W—Cs2^ix^	155.4 (2)
O52^v^—Cs1—O23	87.67 (14)	Mg4—O6W—Cs2^xiii^	135.1 (3)
O10W—Cs1—O23	51.65 (15)	Cs1^v^—O6W—Cs2^xiii^	104.75 (15)
O43^vi^—Cs1—O23	104.71 (17)	Cs2^ix^—O6W—Cs2^xiii^	51.31 (9)
O11^i^—Cs1—O23	145.33 (16)	C1—O11—Mg4	131.9 (6)
O12^i^—Cs1—O23	170.81 (14)	C1—O11—Cs2^ix^	126.5 (6)
O13^i^—Cs1—O23	155.54 (14)	Mg4—O11—Cs2^ix^	94.3 (2)
O22—Cs1—O23	32.67 (13)	C1—O11—Cs1^viii^	72.6 (5)
O21—Cs1—O23	32.57 (14)	Mg4—O11—Cs1^viii^	98.2 (3)
O4W^i^—Cs1—O8W	72.94 (17)	Cs2^ix^—O11—Cs1^viii^	134.2 (2)
O1W—Cs1—O8W	71.44 (16)	C1—O12—Mg1^v^	144.5 (7)
O5W^i^—Cs1—O8W	129.40 (17)	C1—O12—Mg3	92.2 (6)
O3W—Cs1—O8W	125.80 (17)	Mg1^v^—O12—Mg3	123.3 (4)
O6W^ii^—Cs1—O8W	135.59 (17)	C1—O12—Cs1^viii^	71.5 (5)
O8W^iii^—Cs1—O8W	66.15 (18)	Mg1^v^—O12—Cs1^viii^	103.2 (3)
O2W^iv^—Cs1—O8W	137.70 (16)	Mg3—O12—Cs1^viii^	93.7 (2)
O10W^iii^—Cs1—O8W	42.56 (15)	C1—O12—Cs2^ix^	26.0 (5)
O52^v^—Cs1—O8W	176.33 (16)	Mg1^v^—O12—Cs2^ix^	123.9 (3)
O10W—Cs1—O8W	42.13 (15)	Mg3—O12—Cs2^ix^	109.5 (2)
O43^vi^—Cs1—O8W	103.32 (18)	Cs1^viii^—O12—Cs2^ix^	90.21 (13)
O11^i^—Cs1—O8W	92.38 (15)	C1—O13—Mg3	90.8 (5)
O12^i^—Cs1—O8W	98.68 (15)	C1—O13—Cs1^viii^	69.1 (5)
O13^i^—Cs1—O8W	67.61 (14)	Mg3—O13—Cs1^viii^	91.7 (2)
O22—Cs1—O8W	66.01 (14)	C1—O13—Cs2^ix^	72.2 (5)
O21—Cs1—O8W	96.34 (14)	Mg3—O13—Cs2^ix^	150.0 (3)
O23—Cs1—O8W	90.46 (14)	Cs1^viii^—O13—Cs2^ix^	104.35 (14)
O42^vii^—Cs2—O42^viii^	96.5 (2)	Mg2—O7W—Cs2^i^	87.6 (3)
O42^vii^—Cs2—O32^vii^	115.7 (2)	Mg2—O8W—Cs1^iii^	131.9 (3)
O42^viii^—Cs2—O32^vii^	106.4 (2)	Mg2—O8W—Cs1	114.3 (3)
O42^vii^—Cs2—O22	101.4 (2)	Cs1^iii^—O8W—Cs1	113.85 (18)
O42^viii^—Cs2—O22	124.1 (2)	C5—O51—Mg2	93.2 (5)
O32^vii^—Cs2—O22	112.10 (17)	C5—O51—Cs1	156.7 (5)
O42^vii^—Cs2—O53^viii^	69.5 (2)	Mg2—O51—Cs1	110.1 (2)
O42^viii^—Cs2—O53^viii^	62.2 (2)	C5—O51—Cs2^i^	44.7 (4)
O32^vii^—Cs2—O53^viii^	168.44 (17)	Mg2—O51—Cs2^i^	57.54 (17)
O22—Cs2—O53^viii^	75.52 (18)	Cs1—O51—Cs2^i^	151.86 (15)
O42^vii^—Cs2—O11^ix^	168.8 (2)	C5—O51—Cs2^vii^	41.9 (5)
O42^viii^—Cs2—O11^ix^	77.0 (2)	Mg2—O51—Cs2^vii^	60.22 (17)
O32^vii^—Cs2—O11^ix^	58.68 (17)	Cs1—O51—Cs2^vii^	153.67 (15)
O22—Cs2—O11^ix^	89.88 (17)	Cs2^i^—O51—Cs2^vii^	47.04 (6)
O53^viii^—Cs2—O11^ix^	114.14 (17)	C5—O52—Cs1^ii^	163.0 (6)
O42^vii^—Cs2—O53^vii^	61.2 (2)	C5—O52—Cs2^i^	56.8 (5)
O42^viii^—Cs2—O53^vii^	67.60 (19)	Cs1^ii^—O52—Cs2^i^	108.13 (17)
O32^vii^—Cs2—O53^vii^	73.77 (17)	C5—O52—Cs2^vii^	60.2 (5)
O22—Cs2—O53^vii^	161.46 (17)	Cs1^ii^—O52—Cs2^vii^	104.91 (17)
O53^viii^—Cs2—O53^vii^	101.82 (15)	Cs2^i^—O52—Cs2^vii^	50.61 (7)
O11^ix^—Cs2—O53^vii^	107.61 (17)	C4—O41—Mg2	124.5 (6)
O42^vii^—Cs2—O31	67.9 (2)	C4—O41—Cs1^iii^	138.8 (5)
O42^viii^—Cs2—O31	163.9 (2)	Mg2—O41—Cs1^iii^	96.2 (2)
O32^vii^—Cs2—O31	85.09 (17)	C4—O41—Cs2^i^	60.1 (5)
O22—Cs2—O31	58.40 (16)	Mg2—O41—Cs2^i^	73.5 (2)
O53^viii^—Cs2—O31	106.45 (17)	Cs1^iii^—O41—Cs2^i^	152.17 (18)
O11^ix^—Cs2—O31	119.04 (16)	C4—O41—Cs2^vii^	56.7 (5)
O53^vii^—Cs2—O31	106.15 (16)	Mg2—O41—Cs2^vii^	71.9 (2)
O42^vii^—Cs2—O2W	48.3 (2)	Cs1^iii^—O41—Cs2^vii^	149.07 (17)
O42^viii^—Cs2—O2W	118.2 (2)	Cs2^i^—O41—Cs2^vii^	53.02 (8)
O32^vii^—Cs2—O2W	133.11 (17)	C4—O42—Cs2^vii^	145.8 (8)
O22—Cs2—O2W	53.27 (17)	C4—O42—Cs2^i^	123.5 (7)
O53^viii^—Cs2—O2W	58.31 (17)	Cs2^vii^—O42—Cs2^i^	83.5 (2)
O11^ix^—Cs2—O2W	142.97 (17)	C4—O42—Cs1^xiv^	75.4 (6)
O53^vii^—Cs2—O2W	109.42 (17)	Cs2^vii^—O42—Cs1^xiv^	116.4 (2)
O31—Cs2—O2W	48.41 (17)	Cs2^i^—O42—Cs1^xiv^	112.2 (3)
O42^vii^—Cs2—O6W^ix^	120.0 (2)	C4—O43—Cs1^xiv^	140.8 (7)
O42^viii^—Cs2—O6W^ix^	53.5 (2)	C4—O43—Cs2^i^	44.8 (6)
O32^vii^—Cs2—O6W^ix^	53.01 (17)	Cs1^xiv^—O43—Cs2^i^	105.1 (2)
O22—Cs2—O6W^ix^	138.61 (17)	C4—O43—Cs2^vii^	41.1 (5)
O53^viii^—Cs2—O6W^ix^	115.48 (17)	Cs1^xiv^—O43—Cs2^vii^	102.23 (19)
O11^ix^—Cs2—O6W^ix^	48.78 (17)	Cs2^i^—O43—Cs2^vii^	49.58 (9)
O53^vii^—Cs2—O6W^ix^	59.37 (17)	C3—O32—Mg4^xv^	91.0 (6)
O31—Cs2—O6W^ix^	137.44 (17)	C3—O32—Cs2^vii^	141.6 (5)
O2W—Cs2—O6W^ix^	167.16 (17)	Mg4^xv^—O32—Cs2^vii^	94.4 (2)
O42^vii^—Cs2—O9W	108.9 (2)	C3—O32—Cs2	72.2 (5)
O42^viii^—Cs2—O9W	148.0 (2)	Mg4^xv^—O32—Cs2	152.9 (3)
O32^vii^—Cs2—O9W	45.22 (16)	Cs2^vii^—O32—Cs2	112.07 (17)
O22—Cs2—O9W	70.37 (17)	C3—O33—Mg3^ii^	146.5 (7)
O53^viii^—Cs2—O9W	144.84 (16)	C3—O33—Mg4^xv^	91.9 (6)
O11^ix^—Cs2—O9W	74.59 (17)	Mg3^ii^—O33—Mg4^xv^	120.1 (3)
O53^vii^—Cs2—O9W	107.74 (17)	C3—O33—Cs1^ii^	129.4 (5)
O31—Cs2—O9W	47.24 (16)	Mg3^ii^—O33—Cs1^ii^	73.4 (2)
O2W—Cs2—O9W	93.49 (15)	Mg4^xv^—O33—Cs1^ii^	74.3 (2)
O6W^ix^—Cs2—O9W	96.06 (15)	C3—O33—Cs2^vii^	50.4 (5)
O42^vii^—Cs2—O7W^viii^	118.1 (2)	Mg3^ii^—O33—Cs2^vii^	160.5 (3)
O42^viii^—Cs2—O7W^viii^	67.99 (19)	Mg4^xv^—O33—Cs2^vii^	50.09 (17)
O32^vii^—Cs2—O7W^viii^	126.18 (17)	Cs1^ii^—O33—Cs2^vii^	87.18 (11)
O22—Cs2—O7W^viii^	56.80 (16)	Mg2—O9W—Cs2	156.6 (4)
O53^viii^—Cs2—O7W^viii^	49.80 (17)	Mg2—O9W—Cs2^vii^	89.1 (3)
O11^ix^—Cs2—O7W^viii^	68.33 (16)	Cs2—O9W—Cs2^vii^	114.13 (19)
O53^vii^—Cs2—O7W^viii^	135.04 (16)	C5—O53—Mg2	90.1 (5)
O31—Cs2—O7W^viii^	114.68 (15)	C5—O53—Cs2^i^	136.9 (5)
O2W—Cs2—O7W^viii^	85.72 (16)	Mg2—O53—Cs2^i^	105.0 (3)
O6W^ix^—Cs2—O7W^viii^	98.27 (16)	C5—O53—Cs2^vii^	134.6 (5)
O9W—Cs2—O7W^viii^	113.43 (18)	Mg2—O53—Cs2^vii^	109.7 (3)
O42^vii^—Cs2—O9W^vii^	60.4 (2)	Cs2^i^—O53—Cs2^vii^	78.18 (15)
O42^viii^—Cs2—O9W^vii^	114.08 (19)	Cs1^iii^—O10W—Cs1	116.18 (19)
O32^vii^—Cs2—O9W^vii^	55.41 (16)	Cs1^iii^—O10W—Cs2	112.02 (16)
O22—Cs2—O9W^vii^	120.86 (16)	Cs1—O10W—Cs2	118.44 (16)
O53^viii^—Cs2—O9W^vii^	129.17 (16)		

Symmetry codes: (i) *x*−1, *y*, *z*; (ii) *x*−1/2, −*y*+3/2, *z*+1/2; (iii) −*x*+2, −*y*+1, −*z*; (iv) *x*−1/2, −*y*+3/2, *z*−1/2; (v) *x*+1/2, −*y*+3/2, *z*−1/2; (vi) −*x*+3/2, *y*+1/2, −*z*+1/2; (vii) −*x*+2, −*y*+1, −*z*+1; (viii) *x*+1, *y*, *z*; (ix) −*x*+3, −*y*+1, −*z*; (x) *x*+1, *y*, *z*−1; (xi) *x*+1/2, −*y*+3/2, *z*+1/2; (xii) −*x*+3, −*y*+1, −*z*+1; (xiii) *x*, *y*, *z*−1; (xiv) −*x*+3/2, *y*−1/2, −*z*+1/2; (xv) *x*−1, *y*, *z*+1.

### Hydrogen-bond geometry (Å, º)

**Table:**

*D*—H···*A*	*D*—H	H···*A*	*D*···*A*	*D*—H···*A*
O1*W*—H1*A*···O51	0.82 (5)	1.85 (6)	2.658 (10)	172 (12)
O1*W*—H1*B*···O43^vi^	0.83 (5)	1.89 (6)	2.686 (11)	162 (12)
O2*W*—H2*A*···O52^viii^	0.80 (5)	2.01 (6)	2.783 (11)	160 (12)
O2*W*—H2*B*···O42^vii^	0.81 (5)	1.93 (6)	2.734 (12)	170 (13)
O3*W*—H3*A*···O52^v^	0.82 (5)	2.23 (6)	2.993 (12)	157 (11)
O3*W*—H3*B*···O41^iii^	0.82 (5)	1.92 (6)	2.737 (11)	175 (12)
O4*W*—H4*A*···O43^xvi^	0.82 (5)	1.93 (6)	2.724 (11)	162 (13)
O4*W*—H4*B*···O51^viii^	0.82 (5)	1.84 (6)	2.661 (9)	174 (14)
O5*W*—H5*A*···O52^xvii^	0.82 (5)	2.04 (6)	2.853 (10)	169 (13)
O5*W*—H5*B*···O41^ix^	0.80 (5)	2.05 (6)	2.831 (10)	164 (13)
O6*W*—H6*A*···O43^iii^	0.84 (5)	2.21 (6)	3.021 (13)	165 (13)
O6*W*—H6*B*···O52^x^	0.81 (5)	1.98 (6)	2.783 (10)	174 (13)
O7*W*—H7*B*···O23^i^	0.83 (5)	2.07 (7)	2.843 (10)	155 (13)
O8*W*—H8*A*···O10*W*	0.82 (5)	2.02 (6)	2.815 (10)	165 (13)
O8*W*—H8*B*···O10*W*^iii^	0.82 (5)	2.02 (7)	2.815 (10)	164 (15)
O9*W*—H9*A*···O31	0.82 (5)	2.09 (7)	2.869 (10)	161 (13)
O9*W*—H9*B*···O32^vii^	0.82 (5)	1.87 (7)	2.673 (10)	170 (13)
O10*W*—H10*A*···O13^ix^	0.81 (5)	2.01 (6)	2.798 (9)	162 (13)
O10*W*—H10*B*···O22	0.90 (13)	1.84 (13)	2.730 (10)	169 (12)

Symmetry codes: (i) *x*−1, *y*, *z*; (iii) −*x*+2, −*y*+1, −*z*; (v) *x*+1/2, −*y*+3/2, *z*−1/2; (vi) −*x*+3/2, *y*+1/2, −*z*+1/2; (vii) −*x*+2, −*y*+1, −*z*+1; (viii) *x*+1, *y*, *z*; (ix) −*x*+3, −*y*+1, −*z*; (x) *x*+1, *y*, *z*−1; (xvi) −*x*+5/2, *y*+1/2, −*z*+1/2; (xvii) *x*+3/2, −*y*+3/2, *z*−1/2.
