# Supplementary material for: Dicaesium tetra­magnesium penta­kis­(carbonate) deca­hydrate, Cs2Mg4(CO3)5·10H2O
Source: IUCrdata. 2020 Feb 11;5(Pt 2):x200165. doi: 10.1107/S2414314620001650 (PMC9462181; doi:10.1107/S2414314620001650)
Supplement: Supplementary file 4 [file x-05-x200165-sup3.pdf]

Supporting Information

S1 Thermal Analysis

The composition of the title compound was confirmed by thermal analysis (Fig. S1). The released gases were identified by IR-spectroscopy which was coupled with the thermal analysis. The solid residues were characterised by x-ray powder diffraction after the second and the third step of decomposition (Table S1).

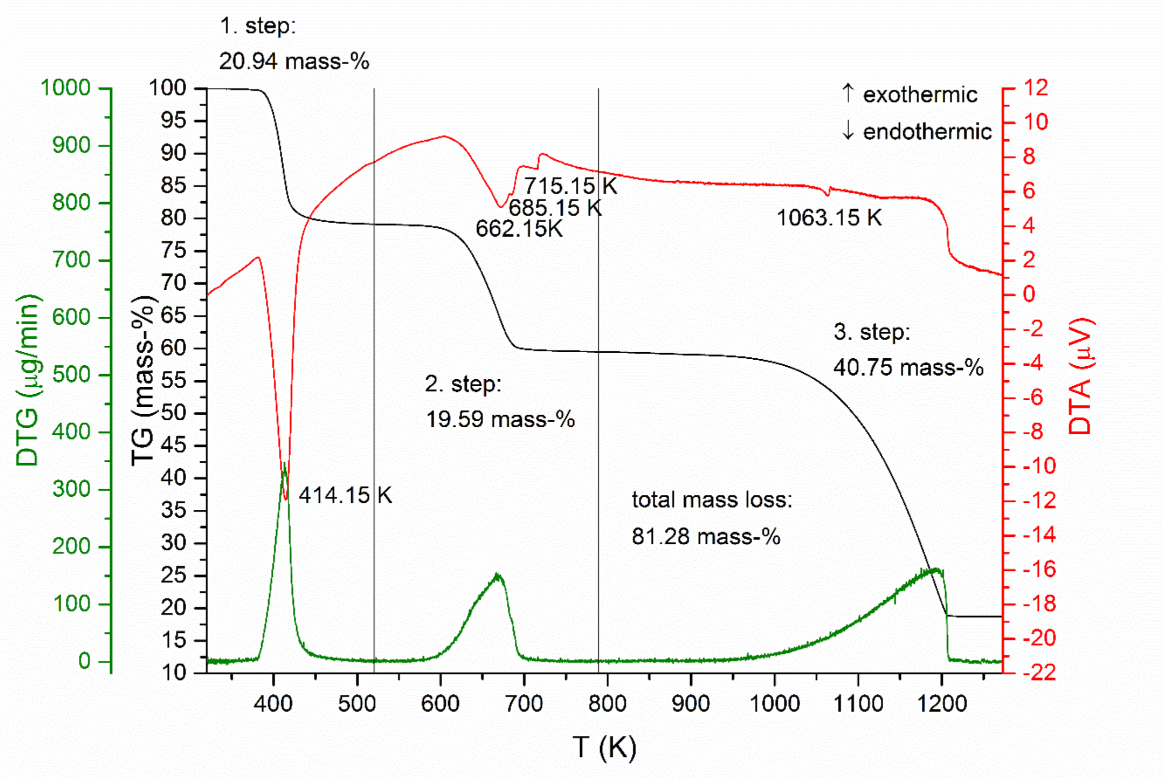

Figure S1: Thermal analysis of $Cs_2Mg_4(CO_3)_5{\cdot}10H_2O$ (TG/DTA 220 instrument of Seiko Instruments, heating rate: 5 K/min, argon flow: 300 $cm^3\ min^{-1}$, reference substance: Al_2O_3).

Table S1: Decomposition of $Cs_2Mg_4(CO_3)_5{\cdot}10H_2O$.

step	temperature range (K)	peak temperatures of the DTA curve (K)	mass loss (%)	theoretical mass loss (%)	released gases according IR spectra	solid residues according XRPD-pattern
1	298 - 523	414.15	20.94	21.36	H_2O	
	reaction of decomposition: $Cs_2Mg_4(CO_3)_5{\cdot}10H_2O(s) \rightarrow Cs_2CO_3(s) + MgCO_3(s) + H_2O(g)$					
2	523 - 789	662.15, 685.15, 715.15	19.59	20.87	CO_2	Cs_2CO_3, MgO
	reaction of decomposition: $Cs_2CO_3(s) + MgCO_3(s) \rightarrow Cs_2CO_3\ (s) + MgO\ (s) + CO_2(g)$					
3	789 - 1273	1063.15	40.75	38.36	melting of Cs_2CO_3 (Liptay, 1976)	MgO
	reaction of decomposition: $Cs_2CO_3(s) \rightarrow Cs_2CO_3(l) \rightarrow Cs_2CO_3(g)$					

S2 SEM

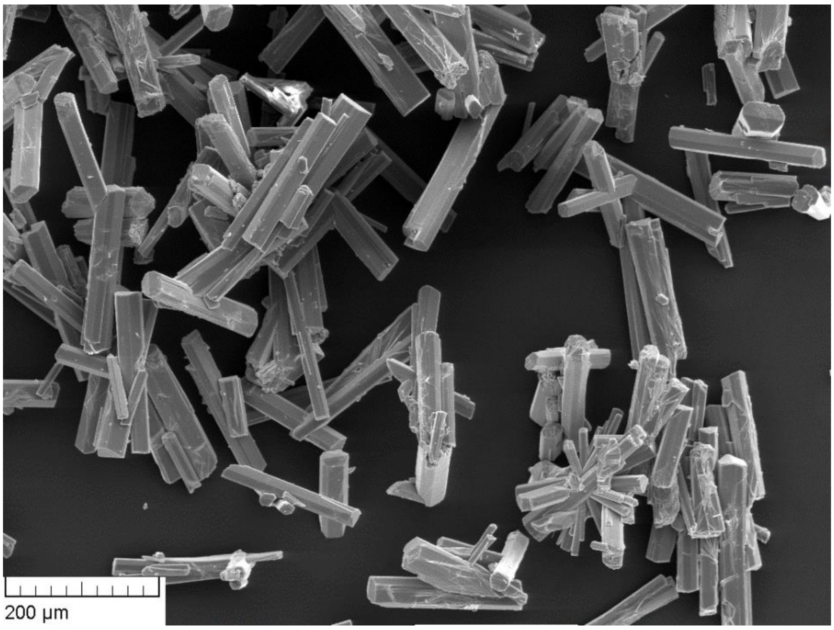

Figure S2: SEM-image of $Cs_2Mg_4(CO_3)_5{\cdot}10H_2O$ (TESCA Vega 5130 SB, accelerating voltage: 20 kV, sample was coating with gold).

S3 Powder X-ray diffraction pattern

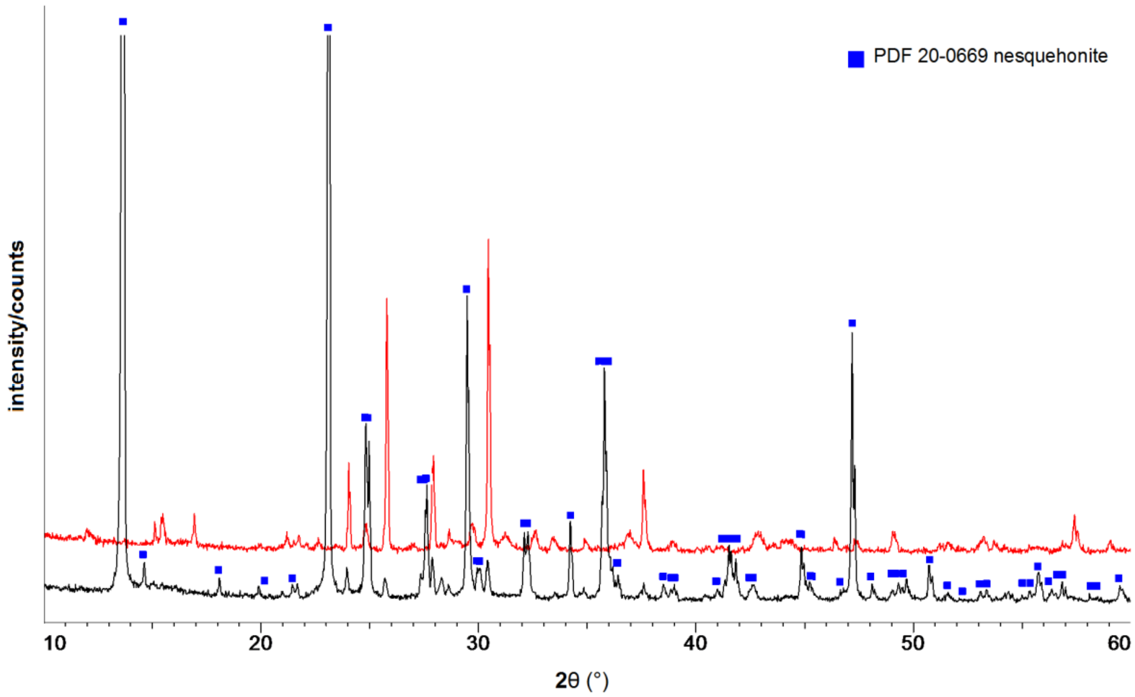

Figure S3: Powder X-ray diffraction patterns of the mixture of $MgCO_3{\cdot}3H_2O$ (nesquehonite) and $Cs_2Mg_4(CO_3)_5{\cdot}10H_2O$ after two days of storage (black line), $Cs_2Mg_4(CO_3)_5{\cdot}10H_2O$ after 16 days (red line) and reference data for $MgCO_3{\cdot}3H_2O$ (blue dots) (Bruker D8 Discover diffractometer in Bragg-Brentano set up, Cu-$K_{\alpha 1}$ radiation, Vantec 1 detector, preparation as flat plate).

S4 IR-spectra

The IR-spectrum of the title compound (KBr) shown in Fig. S4 exhibits the following characteristic bands (Coleyshaw *et al.*, 2003): 3394 cm^{-1} (O-H stretching vibrations of the water molecules), 1675 cm^{-1} (deformation vibrations of the water molecules), 1516, 1389, 1063 cm^{-1} (C-O valence vibrations of the carbonate units), 890, 782, 855 cm^{-1} (deformation vibrations of the carbonate units out of the plane) and 690, 605 cm^{-1} (deformation vibration of the carbonate units in the plane).

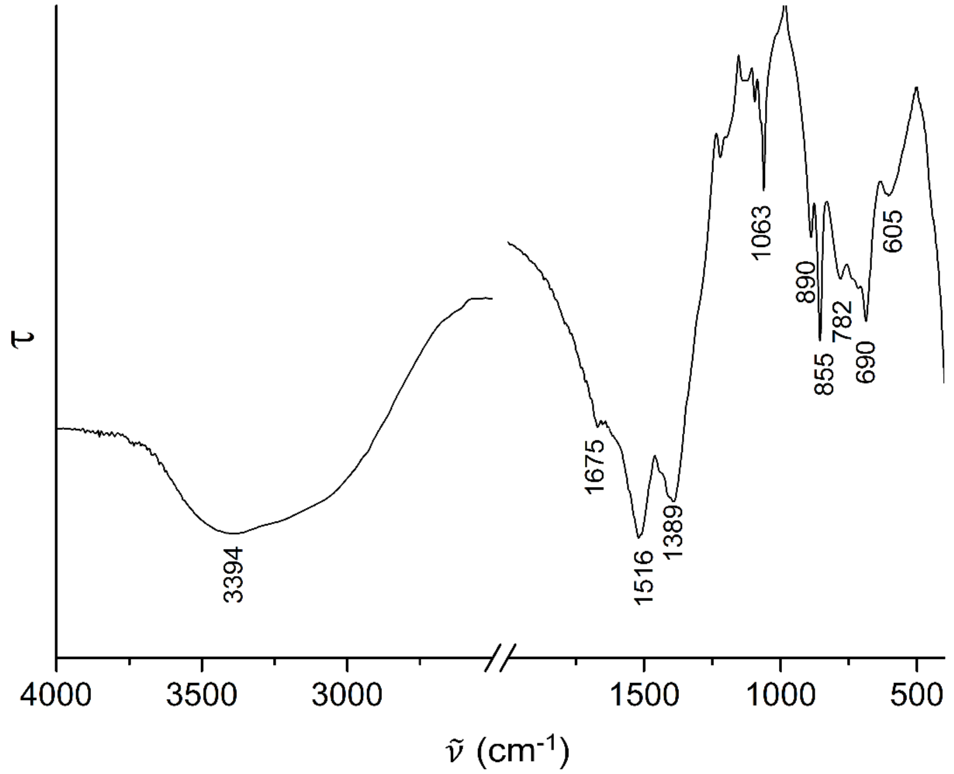

Figure S4: FT-IR spectra of $Cs_2Mg_4(CO_3)_5{\cdot}10H_2O$ at ambient conditions (KBr blank, Nicolet 380X of Thermo Electron Company, DLaTGS-detector)

References

Coleyshaw, E. E., Crump, G. & Griffith, W. P. (2003). *Spectrochim. Acta, Part A*. **59A**, 2231–2239.
Liptay, G. (H.) (1976). Editor. *Atlas of thermoanalytical curves, 5.* Budapest: Akadémiai Kiado.
